# How St. Bartholomew's Hospital Can Be Saved

**Published:** 1904-11-05

**Authors:** 


### The Hospital.

A Journal of

The Medical Sciences and Hospital Administration.

Vol. XXXVII.—No. 945. NOVEMBER 5, 1904.

### How St. Bartholomew's Hospital can be Saved.

The article we published in our issue of the 22nd ult. has brought us strong confirmation of the widespread hope which exists that the resignation of the Treasurer and the Clerk may be made the opportunity to save St. Bartholomew's Hospital. We use the word save advisedly, for it would be the salvation of St. Bartholomew's for the Governors to avail themselves of the present opportunity to adopt a new policy, and so secure a perfect and up-to-date modern hospital without a moment's avoidable delay. The old policy has been tried, and it has failed, as the letter of Sir Trevor Lawrence in the *Times* of the 27th ult. eloquently testifies. The appeal in support of that policy was started in January last, and was backed by the splendid generosity of their Majesties the King and Queen and of the Prince of Wales. In the result some £40,000 only has been obtained since the Mansion House meeting, a fact which makes the fiasco so plain and incontrovertible that it is unique in the history of hospital appeals. It has happened more than once in recent years that the inhabitants of a great provincial city have raised for their hospital upwards of £200,000 in six months or a year. In such circumstances, it would be a waste of words to further discuss this lamentable failure, which we foretold must result from the wrong policy being pursued.

The future of St. Bartholomew's Hospital now rests with the President and Governors, who have a splendid opportunity to show that a wise policy, carefully thought out and directed by the right men, can speedily place our oldest hospital and medical school in the proud position of being the most modern establishment of the kind in the United Kingdom. Let those who are responsible for the present failure have the grace to stand aside, in favour of younger and abler men, who have the experience and the brains to deal with the serious financial questions involved, and to place the finances of St. Bartholomew's Hospital, its buildings, equipment, and administration, once and for all, on a sound basis worthy of the history of so great an institution.

We have been led to call attention once more to the matter by the representations which have reached us from influential quarters, owing to the not unnatural feelings of exasperation which have been aroused by the ineptitude of the policy pursued in the past. It has become apparent in the course of the discussions relating to the affairs of St. Bartholomew's Hospital during the past two years, that an increasing number of Governors, and indeed the whole of the independent Governors who have no axes to grind, are in favour of a new policy, under a new treasurer endowed with the necessary financial qualifications, experience, and public spirit to ensure its success. The Governors are fortunate in having such a man in Mr. Andrew Motion, who was the largest individual contributor to the extension fund, who had formerly served on the house committee of the hospital, and has recently re joined it, and who has displayed, under very trying circumstances, a whole-hearted devotion to its interests. The key to the solution of the difficulty was supplied by Sir Sydney Waterlow, before any of the steps were taken which have resulted in the present fiasco. It appears that St. Bartholomew's is the owner of a number of licensed premises, which have been heretofore let on short leases, whereby the productive power of this property has been curtailed in the past. If the freeholds of this portion of the property were to be taken in hand and realised under a gentleman of the experience of Mr. Andrew Motion, the financial position might be cleared and greatly improved. A policy developed on these lines should enable the new treasurer to formulate a scheme which would provide all the money which is required. Such a plan would make it easy for any additional revenue required for a limited number of years to be raised without much difficulty. It would have the further advantage of modernising the hospital, by making it a public rather than a private corporation as it is at present, which would ensure its periodical inspection, and the establishment of adequate control, making for the highest efficiency in every department of the hospital.

Briefly stated, the position is this. A great institution with enormous funds, the power of which cannot be realised by men who have not been trained in large businesses, and who have not been accustomed, and have not therefore the knowledge to handle great funds, should be under the control of able financiers and administrators. The results which follow an absence of such control are well shown by a statement of Mr. Motion, published in the *Westminster Gazette* of the 27th ultimo. "Our income is about £90,000 a year, but before that is free to be applied to the practical purposes of the hospital it has been diminished in one way and another by £30,000. It seems ridiculous, and I think nothing will meet the present muddle but a properly organised commission of inquiry or a royal commission if necessary." We venture to say that there will be no need for a royal commission, providing the governors of St. Bartholomew's Hospital will now come forward and do their duty by taking the matter in hand, appointing a man of sufficient force of character as treasurer, and so securing the introduction of business methods into the management of one of the finest estates which has ever been possessed by a great charity in the world's history. Of course, if the independent governors will not assert themselves and devote a little time to attending a meeting or two and recording their votes in favour of a new policy, it may be necessary to appoint a royal commission to institute the necessary reforms. We feel confident, however, that the governors have the will, as they undoubtedly possess the power, to promptly do their duty and so save St. Bartholomew's Hospital.

